# Supplementary material for: Lessons from a pandemic
Source: PLOS Glob Public Health. 2022 Jul 22;2(7):e0000404. doi: 10.1371/journal.pgph.0000404 (PMC10021850; doi:10.1371/journal.pgph.0000404)
Supplement: Attachment — Submitted filename: POINT BY POINT RESPONSE TO THE REVIEWER.docx [file pgph.0000404.s001.docx]

Response to Reviewers

| Reviewer’s comments | Responses |
| --- | --- |
| This paper describes a potentially useful model using 3 strata during which infection might take place and 7 parameters to depict 20 different scenarios during various stages of a pandemic to predict the impact of public health interventions instituted to slow or halt the pandemic.  It is quite simple and user-friendly and lacks possibly confusing complexities. In lacking these complexities, the authors have perhaps run the risk of excluding parameters that that would have a significant impact on the output of the model. For instance, wide variations in household size according to rural and urban location or regional variations regions within a country are not accounted for. Age distribution of population which may have impact on susceptibility to infection or degree of contagiousness are also not accounted for in this model. The same can also be said for variations in weather. Nevertheless, the model can still be useful and may be deployed within sub-national units to account for some of these variations if need be.  Notably, the authors expects this model to be used flexibly and with parameters updated as new data emerge throughout a pandemic. This would help to adapt it to different pandemics and not just a COVID-19 or respiratory virus pandemic. A shortcoming I forsee is the suitability for use in the global south where reliable data may be scarce and rely on the same individuals of various disciplines who are deeply involved in the immediate pandemic response to generate such data as may be needed for the 7 parameters used in this study. On the other hand, assumptions from the global north are rarely applicable to the global south as was seen clearly during the COVID-19 pandemic. All of these may hinder the reliability of the model globally.  Overall, the paper is certainly worth publishing but should expand on the aspects discussed above in the discussion and limitations. | Thank you for these constructive comments.  We agree with the limitations mentioned by the reviewer and completed the discussion (see modifications in red). |
| Reviewer #1: Typographical/writing errors:  Page 3 lines 46 and 47: the words 'missed' should be replaced with the word 'lack'  Page 8 lines 131-133: The sentence beginning 'In bold to the end of the title should be removed and place as a subscript after the table  Throughout the manuscript, SARS-Cov-2 should be changed to SARS-CoV-2  Page 9 line 149: Change 'to get a seventh info' to ' to get the seventh piece of information'  Page 11 line 193-194: '....an average of 16.44, respectively of 5.10 other persons...' should be changed to an '.... average of 16.44 or 5.10 other persons respectively....'  The term 'Auto test' should be changed to 'self test' throughout the manuscript  Page 15 line 282: '...some is not be too difficult to obtain....' should be changed to '.... some are not too difficult to obtain....' | We have also corrected all those points (modifications in red in the manuscript). |
